# Cosmopolitanism as an Aspirational Identity: When Normative Ideals Give Rise to Identity Struggles

**DOI:** 10.1007/s10551-025-06081-y

**Published:** 2025-08-04

**Authors:** Patrizia Hoyer, Sierk Ybema

**Affiliations:** 1 Vrije Universiteit Amsterdam, Amsterdam, The Netherlands; 2 Anglia Ruskin University, Cambridge, UK

**Keywords:** Cosmopolitanism, Aspirational identities, Normativity, Cultural others, Identity struggle

## Abstract

While cosmopolitanism is often celebrated for its contemporary ideals of openness toward people from different cultural backgrounds, this paper reveals how and why these same ideals can generate tensions and identity struggles when rigidly applied in everyday cross-cultural encounters. Based on 32 life story interviews with globally mobile professionals who have worked and lived extensively across different countries, we observed and analyzed how these professionals pursued aspirational identity projects aligned with normative ideals of cosmopolitan ‘being’ (‘thinking globally’), ‘doing’ (‘integrating locally’), and ‘becoming’ (‘transforming the self’), when interacting with people from different cultural backgrounds than their own. In their everyday local encounters, this pursuit of cosmopolitan ideals also produced adverse effects: participants experienced resistance to their global mindset, rejections of their integration attempts, and deflections on their path toward self-improvement. These adverse effects created tensions which we theorize as three types of identity struggles: (a) showing tolerance toward intolerance; (b) enduring discrimination in order to fit in; and (c) denying one’s aspired self-image to match the perception of others. We argue that these identity struggles reveal the limitations of aspirational cosmopolitan identity projects that adhere too strictly to normative ideals, as rigid moral scripts around ‘openness toward the other’ can (somewhat paradoxically) restrict the agency of global professionals in their everyday encounters with cultural others. By drawing attention to this limited agency—and the vulnerability it can produce—this paper contributes a new perspective to the literatures on aspirational identities, cosmopolitan identities, and everyday cosmopolitanism, aiming to help bridge the gap in theory and practice between moral principles and lived cross-cultural encounters.

## Introduction

In light of globalization processes, calls have been made for a new kind of relational ethics when engaging in complex intercultural encounters. In response, scholars from different disciplines have time and again turned to the concept of ‘cosmopolitanism.’ which comprises a set of outlooks, values, or dispositions, with ‘openness toward others’ being the most frequently mentioned characteristic. This notion of ‘openness’ has been central to many theoretical as well as empirical investigations of cosmopolitanism (e.g., Appiah, 2006; Delanty & Harris, 2018; Plage et al., 2017; Skrbiš & Woodward, 2007). A ‘cosmopolitan ethics’ is associated with learning about people from cultural backgrounds other than your own, that is, with learning about ‘cultural otherness’ (Skrbiš & Woodward, 2007). This entails values of inclusiveness, respect, and pluralism (Plage et al., 2017; Skrbiš & Woodward, 2013). Appiah (2006: xv) takes this idea even further, framing cosmopolitan ethics as the acknowledgment of obligations toward others and a recognition of the value of human lives in all their diversity. More generally, when taken to an extreme, the cosmopolitan sentiment culminates in the idea of living in a borderless world of free exchanges, enacted through practices that show a commitment to universalism, communitarianism, and selflessness (Skrbiš & Woodward, 2007).

While certainly a well-intentioned concept, we problematize how cosmopolitanism can give rise to aspirational identities driven by normative cosmopolitan ideals—ideals that do not necessarily reflect the lived realities of cross-cultural encounters and, in fact, may be at odds with them, rendering cosmopolitan aspirants vulnerable. With an interest in the cosmopolitan identity constructions of globally mobile professionals, this paper explores how such cosmopolitan ideals—most notably, around what it means to have a global mindset, practice openness and tolerance toward others, and become a ‘better person’ shape these professionals’ aspirational identity constructions. By drawing attention to their actual, everyday experiences of cross-cultural encounters, we aim to pick up on the discrepancies between abstract cosmopolitan ideals and everyday practices, thus contributing to the literature on everyday cosmopolitanism (e.g., Calhoun, 2008; Horst & Olsen, 2021; Moore, 2013; Plage et al., 2017; Pronovost, 2020; Skrbiš & Woodward, 2007). More concretely, we set out to explore the following research question: *How do the cosmopolitan ideals of globally mobile professionals translate into aspirational cosmopolitan identities, and what are the implications of such identity projects for their everyday encounters with (cultural) others?*

Empirically, we explored this research question by analyzing 32 life story interviews conducted with globally mobile professionals, all of whom had worked and lived extensively across different countries and were thus often confronted with questions of identity and belonging (Hoyer, 2025). And indeed, our theoretically informed analysis shows that these globally mobile professionals embraced cosmopolitan ideals—as delineated in the literature—(e.g., Beck & Sznaider, 2013; Delanty, 2006; Nussbaum, 1994, 2010) and translated them into aspirational identity projects. More specifically, we identified their cosmopolitan ideals in the dimensions of ‘being’ (having a global mindset), ‘doing’ (aspiring local integration), and ‘becoming’ (undergoing self-transformation). However, when describing their lived experiences, participants revealed ‘cracks’ in their idealized identity narratives: They reported that their cosmopolitan aspirations had been challenged in their encounters with cultural others. Our analysis focuses particularly on the dissonance that emerged when their good intentions—e.g., to integrate or ‘have a global outlook’—were met with local resistance, resulting in various forms of social exclusion and thereby limiting their agency to pursue an ideal cosmopolitan self-image.

By nonetheless clinging to their aspired cosmopolitan ideals, even in light of these adverse effects, globally mobile professionals experienced different types of identity struggles: (a) the struggle of showing tolerance toward intolerance; (b) the struggle of enduring discrimination in order to fit in; and (c) the struggle of denying one’s aspired self-image to match the perception of others. Considering the implications of these struggles, we infer two core insights. First, in terms of identity, we argue that aspirational identity projects can become stuck, vulnerable, or distorted in lived cross-cultural encounters. Second, with regard to these cross-cultural encounters, we show that rigid adherence to normative ideals of openness and inclusiveness may actually limit people’s agency to respond genuinely to the complexity and intricacies inherent to social and relational dynamics. To ground these claims, we draw on the literatures on aspirational identity and cosmopolitan identity, the former of which explores how idealized identities that people aspire to become produced and reproduced (Koning & Waistell, 2012; Thornborrow & Brown, 2009) and the latter of which includes an examination of cosmopolitanism as a cultural resource for collective identity construction which can partly encompass a process of self-reflection and self-transformation (e.g., Delanty, 2006; Grinstein & Wathieu, 2012; Nowicka & Kaweh, 2016; Petroccia & Pitasi, 2022; Plage et al., 2017; Skovgaard-Smith & Poulfelt, 2018). Next, we aim to bring these strands together by developing an understanding of cosmopolitanism as a tension-ridden, aspirational identity project that unfolds as actors engage in self-reflexive, everyday encounters and experiences of being-in-the-world-together, which in turn shapes how they navigate conflict. In so doing, we contribute to the literature that frames cosmopolitanism as an everyday, enacted practice rather than merely an abstract-philosophical principle (e.g., Calhoun, 2008; Horst & Olsen, 2021; Moore, 2013; Plage et al., 2017; Pronovost, 2020; Skrbiš & Woodward, 2007).

In the following section, we will first briefly review the existing literature on cosmopolitanism with a focus on its identities and normative ideals, then delineate a framework of cosmopolitanism as an aspirational identity. We end the section by sketching the empirical analysis that will ground the main contributions of this paper.

### Cosmopolitanism as a Morally Informed Identity Project

With an increasing portion of the world’s population immersed in global flows and constant motion (Grinstein & Wathieu, 2012), the past few decades have seen considerable growth in scholarship on cosmopolitanism (Skovgaard-Smith & Poulfelt, 2018) and revived discussions about individual and societal responsibilities (Hendershot & Sperandio, 2009). Cosmopolitanism has long been understood as a consciously chosen identity (Nowicka & Kaweh, 2016), often embraced by individuals with a global orientation (Grinstein & Wathieu, 2012). Such cosmopolitan identities are associated with morally informed expectations and social norms regarding how to perform in interaction with others (Lamont & Aksartova, 2002; Nowicka & Kaweh, 2016; Skovgaard-Smith & Poulfelt, 2018), the details of which we scrutinize below to illuminate how such cosmopolitan ideals shape the identity constructions of global professionals and thus impact their everyday encounters with cultural others.

When tracing the roots of cosmopolitanism, it quickly becomes evident that, from the very outset, it was introduced as a moral concept. Ancient Greek thinkers and the Stoics—many of whom were social outsiders to the polis (city-state)—first developed an image of the ‘cosmopolitan’ or ‘citizen of the world,’ to emphasize ideals of equality, inclusion, and a universal community (Maak, 2009). These cosmopolitan values are still relevant today. In the most general sense, cosmopolitanism refers to “the value of taking into account the perspective of the other” (Delanty, 2014: 2; see also Argyrou, 2015) instead of demanding that others should be like us (Moore, 2013). Over the centuries, this understanding has been further refined through various perspectives, most notably the political, social, and critical (for an overview, see Janssens & Steyaert, 2012; Lettevall, 2020), each of which we elaborate on below.

From a political perspective, cosmopolitanism promotes an ethos of common humanity based on an abstract-philosophical commitment to ‘world citizenship’ over all national, cultural, religious, or ethnic affiliations (Nussbaum, 1994). This Neo-Kantian understanding of cosmopolitanism is framed as a moral imperative to detach from existing identities and loyalties in the name of adopting a universal perspective (Nussbaum, 2010). Inseparable from the impact of globalization and its opponents, this belief becomes particularly poignant as a counterbalance to a perceived upsurge in nationalism (Kristeva, 1991; Nussbaum, 1996), including increasing protections of borders and trade zones, and the rise of right-wing parties around the globe (Strenger, 2019). The political cosmopolitan ideal demands that national or local identities should be denied in favor of universal values and a global mindset (Nussbaum, 1996).

From a social perspective, cosmopolitanism extends beyond a global disposition and universal principles of inclusion to emphasize the importance of everyday social processes. Hence, it is also referred to as practical, ordinary, or everyday cosmopolitanism (Hebdige, 1990; Lamont & Aksartova, 2002). With an interest in actors’ practical experiences of living together in a globalized world, such studies have investigated people’s actual ‘encounters’ of cultural difference, thereby distancing themselves from the abstract, idealized image of cosmopolitanism that is promoted in the political perspective (e.g., Calhoun, 2002; Woodward et al., 2008; see also Høy-Petersen & Woodward, 2018). More than just a ‘curiosity for otherness,’ the social cosmopolitan ideal revolves around a readiness for, and devotion to, taking action—for example, by protecting human rights, integrating into and serving local communities, or helping people in need, irrespective of their origin or belonging (Nowicka & Kaweh, 2016).

From a critical (or self-critical) perspective, also referred to as ‘critical cosmopolitanism’ (Delanty, 2006), the concept of cosmopolitanism already explicitly alludes to an identity project by framing itself as ‘the search for self-transformational possibilities,’ including the always-immanent possibility of critical self-reflection and self-transformation. This suggests, by definition, the mutual constitution of ‘self, world and other,’ thereby not only presupposing a willingness to being open to different cultural backgrounds, but also a readiness to change oneself through a reflexive process of cosmopolitan self-fashioning (Plage et al., 2017). The critical cosmopolitan ideal thus shifts the focus from the other to the self. In the following section, we will elaborate on how these ideals—deriving from the political, social, and critical perspectives on cosmopolitanism—may surface in the identity projects of globally mobile professionals as aspirations to be, act as, and become true cosmopolitans.

### Studying Cosmopolitanism Through the Lens of Aspirational Identities

The notion of aspirational identity underlines the idea that people are sometimes best characterized as ‘aspirants.’ Thornborrow and Brown (2009) define aspirational identity as “a story-type or template in which an individual construes him- or herself as one who is earnestly desirous of being a particular kind of person and self-consciously and consistently in pursuit of this objective” (p. 355). The state that people desire is usually higher, better, and nobler than the status quo. Since the question of ‘who we are’ is always also an ethical question (Cunliffe, 2009) of who we aspire to be or become, it is worthwhile to explore both how people ‘perform’ ethical and aspirational identities in their identity talk (Ybema et al. 2009) and how they make ethical choices within specific contexts (Koning & Waistell, 2012). In their aforementioned definition, Thornborrow and Brown (2009) frame aspirational identity as an ideal or expected version of the self. When conceptualizing cosmopolitanism as a form of aspirational identity, its moral appeal is therefore highly relevant.

And indeed, when studying cosmopolitanism as a ‘lived’ and ‘performed identity,’ its perception as a fundamentally moral-ethical identity soon becomes evident (Ong, 2009). Grounded in normative cultural discourse (Skrbiš & Woodward, 2007), the cosmopolitan identity evokes aspirations of how people should (desire to) be. Since cosmopolitanism is ethically associated with the ability to ‘get along’ with people from other cultural backgrounds (Plage et al., 2017), cosmopolitan identities are linked to traits of being conscientious, sensitive to cultural diversity, concerned about rights and equality, and, of course, ‘open to the world’ (Pronovost, 2020). According to Skovgaard-Smith and Poulfelt (2018), this is achieved through the collective mobilization of cosmopolitanism as a cultural resource or ideology, enacted through a process of socially and relationally accomplished identity construction.

In line with the political, social, and (self-)critical ideals prompted by the concept, cosmopolitan aspirations revolve around the pursuit of a positive moral self-image (Papastephanou, 2015) as someone committed to principles of global solidarity, justice, and democracy (Horst & Olsen, 2021), who subordinates self-interest (when required) for the betterment of a larger community. Importantly, this community extends beyond the boundaries of kin and country, and potentially includes the whole of humanity (Moore, 2013). Resting atop a moral hierarchy, such an aspired identity also implicitly conjures up a contrast between the cosmopolitan ‘citizen of the world’ and the ‘non-cosmopolitan’ the latter of whom is assumed to be inward-looking, sedentary, and locally retrenched. Ulrich Beck (2002), one of the most influential figures on cosmopolitan sociology (Argyrou, 2015), tellingly associates the term cosmopolitanism with ‘progress,’ as it evokes an image of metropolitan multiculturalism and mobility—in strong contrast to the non-cosmopolitan, monocultural hinterlands (see also Calhoun, 2008; Pronovost, 2020). As Pronovost (2020, p. 4) provocatively states, we live in a world where “it is better to be considered cosmopolitan than not.”

Framing one’s cosmopolitan identity as inherently positive can influence how people engage in their everyday encounters with others. Skovgaard-Smith and Poulfelt (2018), for example, noted that global professionals, rooted in their cosmopolitan self-identifications, are prone to making ‘us’ versus ‘them’ distinctions—between themselves as ‘citizens of the world’ who show openness and respect for cultural others, and those whom they consider narrow-minded and parochial, because they are locally oriented toward a monoculture. Other scholars have similarly noted that idealized cosmopolitan identity projects can contain contradictions, especially when cosmopolitan values and practices coexist with exclusionary ‘un-cosmopolitan’ expressions. As Høy-Petersen and Woodward (2018) observed, even the most robust, ‘archetypically’ ethical cosmopolitans will suspend their repertoires of ethical openness temporarily, for example, when managing experiences of threat. These observations led to their conclusion that cosmopolitan idealism does not always pass the ‘real-life test.’

Building on these critical studies—which show how cosmopolitan identity constructions can foster a sense of moral superiority and/ or falter in real-life interactions—this study examines how the normative ideals of political, social, and critical cosmopolitanism inform individuals’ aspirational identities and shape their everyday encounters with cultural others. Now turning to our empirical analysis, we thus proceed from the assumption that unreflective adherence to an idealized, aspirational self-image can complicate how people engage with cultural others in everyday situations.

## Methodology

### Sampling

We conducted 32 interviews with 26 globally mobile professionals from both academic (16) and business (10) contexts to empirically investigate the research question: *How do the cosmopolitan ideals of globally mobile professionals translate into aspirational cosmopolitan identities, and what are the implications of such identity projects for their everyday encounters with (cultural) others?* Of our 32 interviews, six were follow-ups. One interviewee from academia held a hybrid position—an outlier, but interesting to include in the sample—as he also worked for a governmental organization at the time of the interview (and in the years leading up to it). Although global career mobility may be characteristic of a wide range of occupations, this study specifically focuses on the contexts of academia and business. We chose these two distinct work contexts because they were different enough to ensure that our observations would not be confined to a singular environment, yet similar enough to allow for meaningful comparison across cases. As we zoomed in on how globally mobile professionals constructed their aspirational cosmopolitan identities, our impression of interviewees’ responses on this subject did not differ across the contexts. We hence decided to draw on all 32 interviews as one collective sample. After providing a short overall framing, the remainder of this section will point out some of the differences and similarities across the academic and business contexts.

#### Overall

Generally speaking, members of both academia and the business world display a moderate degree of global mobility, with some showing more extensive ‘hyper-mobility’ (Cohen & Gössling, 2015). At the same time, mobility is not inherent to the nature of these occupations, as is the case with diplomats and humanitarian aid workers. Instead, mobility remains a choice that is often driven by individuals’ own lifestyle and career considerations (Hoyer, 2025). In the following paragraphs, we will elaborate on mobility practices within both contexts.

#### Academia

The (mass) global mobility of academics goes far beyond the traditional overseas sabbatical and may be attributable to the globalization of the higher education sector, where new public management schemes prompt universities to compete internationally for outstanding scholars in order to secure their place in the rankings. At the same time, many academics are facing deteriorating working conditions in their home country, including the lack of opportunities for tenure or promotion, a growing number of short-term contracts, and the detrimental effects of job insecurity (Forster, 2001; Miller, 1995). These push-and-pull forces often trigger academics to explore career opportunities outside their own home country institutions (Richardson & Zikic, 2007).

#### Business

In the business context, people are also increasingly seeking positions abroad. More generally, globalization and the advent of free trade areas have led to a new and intensifying landscape of global competition between firms. As such, the ability to develop globally competent managers and leaders has become a highly ranked priority for business development (Suutari, 2003). Not surprisingly, then, international work experience has become an important way for business professionals to develop their career capital (Dickmann & Harris, 2005). And indeed, when going abroad, these globally mobile professionals are often given new roles which are associated with greater responsibilities, prestige, and career opportunities (Harvey, 1985; Shaffer et al., 2001).

#### Shared Experience

To investigate how globally mobile professionals make sense of their encounters with people from different cultures, this study focuses on the career paths of people who have lived and worked extensively in countries outside of their country of origin. More specifically, while there are many alternative forms of international work (Mayrhofer & Reiche, 2014)—including business travel (Welch et al., 2007), virtual assignments (Maznevski et al., 2006), and short-term transfers (Tahvanainen et al., 2005)—this study takes a particular interest in the global mobility of professionals who, rather than being assigned to expatriate roles, actually self-initiated their work assignment abroad and relocated their residence from one country to another—several times—in pursuit of their career ambitions.

On average, the people we interviewed for this study had lived in more than four countries outside their country of origin, with durations of stay in any one host country ranging between 6 months and 19 years, and averaging slightly less than 3 years. Several interviewees reported efforts to learn the local language (if this was not English), depending on factors such as their expected length of stay, their own perceived language-learning skills, and their willingness and ability to immerse themselves in the local context. Of the 32 interviewees, 12 self-identified as male and 14 as female. The (convenience) sampling was based on a snowball system, where first contacts were established through the first author’s own social network, while further contacts were established through referrals from interviewees.

Research participants came from 18 countries of origin across four different continents. Concerning their socio-economic background, some interviewees came from a working class background, the majority had a middle class background, and only a few were born into families in which their parents had been highly mobile as part of an elite global career path. Overall, all research participants arguably inhabited a position of privilege due to having been university educated and able to pursue a (global) career (Hoyer, 2020). Interestingly, interviewees often reported that their first country relocation had been motivated either by a ‘good opportunity’ abroad or a desire to explore a new country, but that this experience had created a certain pull that led them to subsequently move from one country to the next.

Table 1 provides an overview of research participants, while Table 2 lists the participants’ countries of origin and/or childhood residence, as well as their various host countries (34 in total). In order to guarantee the anonymity of research participants (especially relevant to the participating academics, within a small academic community), all names used in this paper are pseudonyms and the links between interviewees and their host countries are not made visible.

**Table 1:** Overview of research participants

Pseudo name	Age	Working context	# of countries abroad	Total # of years abroad
Paul*	32	Business	3	9
Sophie*	36	Business/Academia	7	10.5
Elitsa*	29	Academia	3	7
Amit*	36	Business	5	11
Tutul	54	Academia	3	24
Emma	52	Academia	4	10
Matteo*	34	Academia	4	11
Martin*	31	Academia	3	9
Luuk	31	Business	3	6
Mia	35	Business	4	11
Sarah	47	Academia	5	17
Finn	35	Business	5	11
Jana	38	Business	4	11
Christoph	35	Academia	7	8.5
Faria	34	Academia	4	8.5
Tina	33	Academia	4	9.5
Isabell	32	Academia	4	8
Mira	35	Academia	3	6
Adnan	34	Academia	4	10
Zarbeen	44	Academia	3	10
Rahul	56	Academia	3	28.5
Annelies	30	Business	5	8.5
Nabil	35	Business	3	5.5
Andreas	38	Academia	5	7.5
Ida	36	Business	5	3.5
Jukka	52	Academia	4	8

^*^ Participants who have been interviewed twice

**Table 2:** Overview countries of origin/upbringing and c﻿o﻿u﻿n﻿t﻿r﻿i﻿e﻿s of relocation

Countries of origin/ upbringing	Countries of relocation
Austria, Bangladesh, Belgium, Brazil, Bulgaria, Canada, England, Fiji, Finland, Germany, India, Italy, New Zealand, Qatar, Singapore, Sweden, Switzerland, The Netherlands	Afghanistan, Argentina, Australia, Belgium, Brazil, Cameroun, Canada, China, Columbia, Cyprus, Denmark, England, Finland, France, French Congo, Germany, Greece, Ireland, Israel, Japan, Mexico, New Zealand, South Africa, Russia, Singapore, Sint Maarten, Sweden, Switzerland, The Netherlands, The Gambia, USA

### Data Collection

For this study, a total of 32 semi-structured interviews were conducted with 26 globally mobile professionals. This included six follow-up interviews with participants who moved countries during the course of this study (mostly tracked via social media) to also capture this new experience. Although it would have been interesting to interview all research participants twice, we limited our follow-up interviews to those who were actively resettling in a new country, assuming that the emotional intensity of such a relocation—and the reflections it might trigger—would offer the most insight into how global mobility shapes one’s sense of self and everyday interactions. All interviewees gave informed consent for the interviews to be audio-recorded and analyzed for this study. The length of interviews ranged from 43 to 128 min, with an average of 83 min. Interviewees were asked to share the ‘story’ of their global journey in some detail. Apart from questions about their motivations for seeking out cross-cultural experiences throughout their career, we also asked interviewees to describe their everyday encounters with others within their local and global communities, and to reflect upon these encounters. Likewise, we asked interviewees about their understanding of, and associations with, the notion of ‘cosmopolitanism’ and to what extent they identified themselves with this term.

### Data Analysis

For the purpose of this paper, a narrative analysis was conducted of the reported career and life journeys of mobile professionals. Narratives are considered particularly well suited to capturing richness and ambiguities—qualities that become especially visible during moments of career transition, as exemplified in cases of global mobility (Hoyer, 2025; Mallett & Wapshott, 2012). As personal career narratives (Christensen & Johnston, 2003) are constantly assembled, refined, and embellished, identity in a narrative framework is seen as mutable and repeatedly up for redefinition (e.g., Fraher & Gabriel, 2014; Ibarra & Barbulescu, 2010; Whittle et al., 2009). At the same time, career stories may help people achieve narrative order, namely by providing a sense of plausibility and continuity across shifting contexts (Hoyer, 2020; Brown et al., 2009), as exemplified by frequent international relocations. The remainder of this section describes how we approached our narrative analysis of the interview material.

As a first analytical step after fully transcribing the interviews, all interview passages where people reported and reflected upon their global journeys were highlighted, including their experience with diversity and other cultural customs in their everyday encounters. This included their experiences of relocations and their ease or struggles to adapt. The interview passages that were highlighted covered a broad range of experiences and sentiments, including: the excitement of learning about a new country and different cultural perspectives, the desire and (in)ability to integrate into a host country community, the frustration of encountering narrow-mindedness from locals, and various experiences of social exclusion and racism.

In a second step, these interview passages were coded using an open-coding-like process (Turner, 1981), generating 51 codes. Third, these codes (and related interview passages) were organized thematically around five main topics: experiences of cultural difference, local integration, encounters with locals, associations with cosmopolitanism, and experiences of personal development. To shift from this first round of content analysis to a more focused narrative investigation of our specific research interest, the thematically ordered interview passages were then systematically analyzed and interpreted following an iterative process of moving between the literature and our interview material.

Given our interest in the notion of cosmopolitanism and its various conceptualizations in the literature, we (abductively) observed how interviewees used their career narratives to enact a number of cosmopolitan ideals, noting that ‘openness toward the other’ took different forms. Our initial observation concerned interviewees’ idealization of their ‘global perspective’ which they framed as their own outlook or ethos for how to ‘be in the world.’ This observation very much aligns with the abstract-philosophical ideals of cosmopolitanism found in the literature (Nussbaum, 1994, 2010). Second, we noted that interviewees had widely credited their openness toward the other as having informed their actual ‘everyday doings’ when engaging with cultural others. Often, these doings were embedded in their desire to locally integrate in their host countries, reflecting a sense of respect for cultural particularities and humbleness on their part. These descriptions resonated with depictions of ‘everyday’ and ‘mundane’ cosmopolitanism in the literature (Beck & Sznaider, 2013).

Lastly, we noted that interviewees often saw their global journeys as opportunities for personal development and growth, enabled through their global journey as a way of ‘becoming a better version of oneself.’ These aspirations align with the cosmopolitan ideal of undergoing ‘self-transformation,’ again as described in the literature (Delanty, 2006). By bringing together these observed patterns in the interview material and mapping them along the existing literature, we noticed a threefold idealization of cosmopolitanism among interviewees, namely: around cosmopolitan ‘being,’ ‘doing’ and ‘becoming.’ Furthermore, we sensed that, by engraining these cosmopolitan ideals in their career and life stories, mobile professionals had been able to construct a self-image we refer to as “the aspirational identity of being a good cosmopolitan.”

Then, with a particular interest in how these cosmopolitan ideals might have clashed with interviewees’ actual everyday encounters with others, we went back to the interview material and paid close attention to the mobile professionals’ reports of challenges, frictions, or disappointments in their everyday interactions. We often observed that such conflict or frictions were attributed to the cultural other, who, in their telling, did not share their global outlook, sabotaged their attempts to locally integrate, or hindered them from becoming their aspired cosmopolitan self. Interestingly, while these frustrating encounters could have created space for difficult yet meaningful negotiations around cultural differences, conflicting values, or contested subject positions, we instead observed a recurring pattern: the globally mobile professionals held on to their idealized self-image—of being a ‘world citizen,’ ‘humble visitor’ or ‘self-optimizer,’—which, then, gave rise to three types of identity struggles, as outlined in the findings. Table 3, at the end of the Findings section, provides an overview of interviewees’ enacted cosmopolitan ideals, how these clashed with their real-life experiences, and the resulting identity struggles. The implications of these identity struggles for people’s aspired identities and their encounters with cultural others will be elaborated on in the Discussion section.

**Table 3:** Aspirational cosmopolitan identities and their related identity struggles

*Perspective on cosmopolitanism and associated ideal*	*How ideals shape aspirational identities*	*Identity struggle*	*Short description*
Political perspective: Ideal of a global mindset and detachment from local identities	Appeal to cosmopolitan ‘being’: think globally	Showing tolerance towards intolerance	Clinging to aspirational identity of open-minded ‘world citizen’, despite clashing worldviews and intolerance
Social perspective: Ideal of integrating in and serving local communities	Appeal to cosmopolitan ‘doing’: integrate locally	Enduring discrimination in order to fit in	Clinging to aspirational identity of ‘humble visitor’ who avoids conflict even in case of social exclusion
Critical perspective: Ideal of critical self-reflection and self-transformation	Appeal to cosmopolitan ‘becoming’: transform yourself	Denying one’s aspired self-image to match the perception of others	Clinging to aspirational identity of ‘selftransformed cosmopolitan’ who sacrifices desired self-image to please others

## Findings

When reporting on their global careers, research participants engaged in a variety of narratives through which their cosmopolitan experience emerged as multifaceted and fraught with tensions, especially when their cosmopolitan idealism clashed with everyday practice. Before describing the tensions that emerged around their failed or faltering aspirations and real-life encounters, we will first sketch the cosmopolitan ethics that infused their talk of self.

Cosmopolitan norms and ideals clearly infused and inspired the identity narratives of the participants in our study, all of whom had embarked on a global career. In line with the dominant discourses around ‘world citizenship’ (Nussbaum, 1994, 2010), ‘everyday cosmopolitanism’ (Beck & Sznaider, 2013) and ‘personal transcendence’ (Delanty, 2006) that can be found in the literature on cosmopolitanism, global professionals fully endorsed and celebrated the aspirations of transcending national borders, embracing otherness and seeking self-improvement. In fact, they seemed to model their cosmopolitanism around three defining aspirations, which we refer to as (1) cosmopolitan ‘being’—aspiring and asserting to be, in essence, a true cosmopolitan (an “I am” statement), i.e., assuming a broad global perspective instead of having a narrow national focus; (2) cosmopolitan ‘doings’—aspiring and asserting to act out and practice cosmopolitanism (“I do”), i.e., desiring both integration into foreign local communities and familiarization with the cultural other; and (3) cosmopolitan ‘becoming’—aspiring and asserting to develop cosmopolitan qualities (“I will be”), i.e., learning from cultural plurality and undergoing a process of self-transformation. These three modes roughly correspond to dominant strands in the literature—specifically, the abstract-philosophical, practical, and critical cosmopolitanisms—and appear to have shaped our interviewees’ aspirational identity projects. These three dimensions also align with popular cosmopolitan expressions such as ‘think globally’ (being), ‘integrate locally’ (doing) and ‘transform yourself’ (becoming).

### Cosmopolitan Idealism and Aspirational Identities

#### Cosmopolitan Being: Think Globally

When claiming to *be* cosmopolitan and to advocate for the cosmopolitan ideal of thinking globally, interviewees stressed an aspiration to have ‘the right attitude’ which is not aligned with parochial or national attachments, but instead seeks ‘world citizenship’. This ‘disposition’ is often found in normative-philosophical framings of cosmopolitanism (Nussbaum, 1994, 2010). The interviewed global professionals typically claimed that they had acquired this disposition in early childhood and/or that they had adopted this attitude over the course of their global career. Participants who had already been exposed to cross-cultural experiences in childhood often reported having developed an appreciation for cultural differences early in life. Importantly, many interviewees who had been less exposed to cross-cultural experiences during childhood still expressed a similar early fascination with cultural plurality.

One particularly striking remark, which was somewhat exceptional yet worth mentioning for its powerful imagery, came from an associate professor who claimed she had been ‘born’ with a global disposition, even though this had not been specifically fostered by her childhood environment. Explaining why she had spent her life consciously seeking out international experiences, Sarah, who had lived in the US and several European countries, laid claim to being cosmopolitan by referring to both her innate disposition and adopted attitude:

I wanted to work with international issues. That international perspective—it’s almost as if it was in my mother’s milk. And once you have lifted your eyes above the borders, you can’t go back. Then working with [national] issues just felt too small for me. (Interview 17)

Despite a quiet upbringing in rural Scandinavia, Sarah saw her ‘global disposition’ as having been metaphorically ‘given to her’ through her mother’s milk and further strengthened later in life. She juxtaposed the cosmopolitan ideal of transcending (artificial) national borders—‘lifting your eyes above the borders’—with national issues, which she deemed ‘small’ and thus less relevant.

In a similar vein, other interviewees also emphasized having adopted a cosmopolitan mindset over the course of their global journey—i.e., the desire and appreciation for cultural plurality. Finn, for instance, explained how a whole ‘new world’ had opened up to him upon joining a global project at the European Organization for Nuclear Research (CERN) as part of his physics education:

The sampling of different cultures is the most important thing. You actually get to see other cultures, how they deal with problems. I’ve learned to sample the best things, that’s really the gold mine. You know, there are different ways to deal with things and you can choose. So, you become very flexible. (Interview 18)

Finn endorsed a flexible, cosmopolitan mindset by framing exposure to cultural plurality as a ‘gold mine,’ something worth striving for. Jana, a business consultant, also positioned herself as a strong proponent of ‘openness toward the other’:

This internationality helped me see that there is no right and wrong. I’ve experienced different societies, different concepts, different ways of thinking. And society forces you into believing: “This is right, this is wrong. It’s black or it’s white.” But it’s not like that and I’ll always stand up for that [belief]. (Interview 19)

Jana’s endorsement of openness, tolerance, and suspending judgment led her to claim that she would actively counter instances of observed intolerance or paternalism, which leads us to the next section on aspirations of ‘cosmopolitan doings,’ which build on the aspirations of cosmopolitan being.

#### Cosmopolitan Doings: Integrate Locally

While having a global mindset is generally framed as an ideal cosmopolitan ‘disposition’ or ‘attitude’ in the literature (Nussbaum, 1994), the ‘everyday’ (Plage et al., 2017) and ‘mundane’ (Beck & Sznaider, 2013) framings of cosmopolitanism focus especially on actors’ daily encounters with local others and idealized acts of local integration. The globally mobile professionals interviewed for our study also reflected this aspiration of ‘cosmopolitan doings’ through their narrative accounts of the conscious, proactive efforts they had made to integrate into local communities and adapt to local customs. This was articulated, for example, by Amit, a business manager from the Global South who had come to Europe for an international MBA program after several years of business projects across different countries:

In the MBA circle, it was all [internationals]. In my free time, I wanted to surround myself with a completely different set of people, so I joined the student theater. For me, those were the real people of the place, because they were mostly [locals]. It’s just my curiosity—to see how these people live, what their life is like. That’s what I still seek when going into a new culture, to become part of that culture. I want to live that life. (Interview 5)

Having lived in five different countries outside his country of origin and portraying himself as fascinated by cross-cultural experiences, Amit described having developed an aspirational self-image of being curious and culturally engaged. In this same interview passage, he further suggested that this cosmopolitan mindset (‘being’) also encouraged him to engage in cosmopolitan practices (‘doing’), namely, his careful choosing of whom to interact with in his free time. He emphasized that he did not desire to be part of an international community far removed from local particularities. Instead, he claimed to actively seek integration into a local community by joining the student theater, where the local language was spoken, which he was still learning at that time.

In a similar vein, Nabil, another business manager from the Global South, described having taken a proactive approach to integration by trying to understand the European context to which he had relocated. He held a strongly normative view about how to behave when moving to a new country:

If we’re talking about integration, I think integration is up to the foreigner, not the locals. People have a very misguided idea about this. If you go to [a country] and start behaving obnoxiously—obnoxious in [local] terms—then of course you will struggle to integrate. […] Even if you don’t drink, there’s no harm in going to the pub or a disco. Part of integration is investing a lot of time in understanding the [local] conversations. I think I’ve done a bit of that. (Interview 29)

Nabil had a clear idea of what it means to be a ‘good’ foreigner (cosmopolitan ‘being’), namely, to care about integration and be willing to understand local customs and conversations. He went on to explain that this also requires an investment in ‘doing’ things differently, such as going to a pub even with a Muslim background and making an effort to understand the locals’ point of view. Essentially, he suggested that cosmopolitan ideals should be realized in everyday encounters and claimed to have “done (a bit of) that.”

Reporting on their efforts to ‘do as the Romans do, when living in Rome,’ interviewees also positioned themselves as ‘humble’ and ‘unassuming.’ Tina, an assistant professor who had previously lived in Australia and several European countries, saw herself as a migrant worker in every host country:

You should try to learn the [local] language, otherwise integration will always be limited to people who are like you, who speak English. But then you’re missing out on getting to know people who are different from you. I’m a social sciences researcher, so of course I want to know people who are different from me. I want to understand. (Interview 22)

Tina’s aspirational identity construction was not just grounded in an abstract ideal of what people ‘should be doing.’ As she told it, her commitment to local integration as an expression of everyday cosmopolitan ‘doing,’ was underlined by, for example, having defended her PhD in a language other than her mother tongue or English and having repeatedly enrolled in classes to learn the language of her current host country.

#### Cosmopolitan Becoming: Transforming One’s Self

In their career narratives, the interviewed globally mobile professionals also endorsed the cosmopolitan ideal of ‘self-transformation,’ which rests upon the aspiration that cosmopolitan ‘being’ (thinking globally) and ‘doing’ (integrating locally) will eventually allow people to become a better person. Especially the notion of ‘critical cosmopolitanism’ (Delanty, 2006) emphasizes the importance of reflexive learning and personal transformation as part of a cosmopolitan ethos. In line with this ideal in the literature, interviewees expressed that their global path had increased their self-awareness and enabled their transformation into a better self. As part of their reflexive process, the participants framed hardships encountered during their global journeys as having been unique learning opportunities. For example, Mira, a senior lecturer of Asian descent who was born and raised in an Arab country before moving to North America and Europe for her university education and first academic position, reflected on the challenges of her career path through a resilience lens:

Moving around has made me grow. Each move was difficult, but it taught me so much—whether it was a new cultural context or learning how to be more resilient. Whether it meant bowing down or not. I used to be quick to speak up in [that country], but you learn to change, to adapt. (Interview 24)

According to Mira, personal growth can thus take different forms in different contexts. Sometimes the local context required her to “bow down” and be more tempered in her responses, such as speaking up and challenging certain behaviors. In her aspiration to become cosmopolitan, she labeled ‘difficult’ cross-cultural experiences as valuable ‘lessons learned.’

Another frequently reported ideal of ‘cosmopolitan becoming’ was that of increased self-awareness: interviewees claimed that their global journey had made them more reflexive about themselves, their own ‘cultural luggage,’ and about ‘who they really are.’ In the words of one participant, Ida:

The more I traveled, the clearer I got about myself, about my strengths and weaknesses, and also my values—which helps a lot. As does acknowledging the fact that some of these might have derived from [my cultural upbringing]. And I was thinking, maybe I shouldn’t be like that, but I also see the advantages of it. It’s really helped me become myself and to feel more settled. (Interview 31)

Having lived in five different countries outside her country of origin, Ida thus described her global journeys as having shaped her sense of self by helping her gain awareness of her strengths, weaknesses, and values, which, according to her, were strongly shaped by her upbringing. While this increased self-awareness occasionally led to self-doubt (“maybe I shouldn’t be like that”), in the end, she concluded that her global path had helped her ‘become’ herself, and thus be more ‘authentic,’ as she later said in the interview.

Other interviewees also explicitly credited their global journey with helping them become a better version of themselves. This was true for Paul, a business manager who had lived in several countries across multiple continents:

I’m open to new things, meaning also to changing my views. Rather than saying: “Well, these are my views, this is how I live, this is how I think,” I’ve changed too. […] This willingness to change made [my journey] quite easy. (Interview 1)

Paul expressed a conviction that international travel requires the willingness and ability to revise one’s views, habits, and beliefs—describing a willingness to “change” who you are and ‘become a new version of yourself’ by seeking out new experiences and trying to understand the perspectives of others in everyday encounters. While the above examples of cosmopolitan ‘being,’ ‘doing’ and ‘becoming’ in people’s aspirational identity projects indicate harmonious co-existence with people from other cultural backgrounds, the following sections will focus on tensions observed between the pursuit of these cosmopolitan ideals and actor’s everyday encounters with cultural others.

### Cosmopolitan Aspirations and Their Related Identity Struggles

#### The Aspirational ‘World Citizen’ Identity: A Clash Between Principles

As demonstrated above, globally mobile professionals aspire to be open-minded world citizens who think globally and embrace cultural plurality. Nonetheless, and as we will now explore, their ideals clashed in their everyday cross-cultural encounters with others who did not share their cosmopolitan mindset. This was often palpable when interviewees, whose global journeys had “broadened their horizons” (as many of them reported), described their attempts to share their new perspectives—sometimes more subtly, sometimes more bluntly—with their local communities. Especially when returning to their home countries after a long global journey, mobile professionals experienced feeling that their family members or childhood friends had not, in their eyes, broadened their “provincial” or “conservative” views:

Sometimes, with some of them, I really think: why are we still good friends? We’re really different politically, [we have] different worldviews. But we still connect on a different level, I guess. […] I always try to stay open to other perspectives as well—to stay a bit flexible. And I’m not too keen on political discussions anyway. When I go back, it’s nice to talk to them—but I am generally happy that I developed new perspectives. (Interview 23)

Isabelle, an academic who had moved countries several times across three continents, described regular visits to her hometown in Austria, where she would meet up with her friends from high school. While she reported enjoying these encounters, she also described her friends’ inward-looking, nationalistic sentiments as clashing with her global mindset. In her striving for openness toward the other, she aimed to suspend judgment on, in her eyes, their narrow-minded views and intolerance. Likewise, Amit experienced limitations when trying to share his new, globally inspired insights with his extended family in India:

I grew up in a conservative environment where everything had to be done a certain way. After talking to people from other cultures, I started questioning these things—like the culture of arranged marriage and a dowry. So, my aunts told my cousins: “Don’t talk to Amit. He’ll put ideas in your head.” So, they stopped talking to me. I was trying to broaden their horizons, but then I realized they really don’t want that. That was another thing I learned. I can have my opinion, but I have no right to impose it on others. (Interview 5)

Amit’s experience with people from other backgrounds changed his perspective on the customs and norms of his home culture, which he had previously taken for granted. His family was not open to his new insights, however, and avoided conversations with him. Interestingly, rather than being upset, their reactions appealed to another cosmopolitan ideal he held about accepting and respecting cultural peculiarities and boundaries.

These illustrations show how, after an extended period abroad and with newly acquired cosmopolitan principles, mobile professionals may no longer be aligned with their old ‘home community’ and culture, which, to them, may have become strange and alienating. Interestingly, the very same cosmopolitan principle of openness toward the other—which calls for a global mindset but also for the suspension of judgment—puts global professionals in a kind of moral split. Or, as we frame it, it elicits the paradoxical identity struggle of ‘showing tolerance toward intolerance.’ Holding on to their ideal of ‘openness toward the other,’ both Isabelle and Amit refrained from ‘educating’ their family and friends about their cosmopolitan insights and ideals, thereby allowing others to be, in their view, ‘un-cosmopolitan’ according to their standards.

Other interviewees recalled similar encounters in their adopted host countries. Despite adhering to the ideal of ‘cosmopolitan being,’ they described feeling disillusioned when confronted with ‘un-cosmopolitan’ responses. Ida, for example, who had prided herself during the interview on her cosmopolitan attitude, nonetheless acknowledged a downside:

I always tell myself: “Take the time to observe first, understand first, before judging.” People tend to judge easily. And that’s where I'm very, very cautious. […] Sometimes I’m even overly tolerant, because if somebody’s behaving really, really badly, I always try to understand: Why is he doing it? Sometimes I should just say, “That’s bad and I don’t accept it.” Sometimes I'm showing too much empathy. Because I am always questioning and trying to understand. (Interview 31)

In this interview passage, Ida, who managed a culturally diverse team at a large multi-national company, was of two minds. She was both holding on to her cosmopolitan ideal of trying to understand different cultural customs as part of her aspirational identity project and expressing a sense of doubt: is her tolerance always justified, especially when someone appears to simply be behaving “badly” and possibly taking advantage of her “overly tolerant” mindset? This identity struggle, of whether to tolerate intolerance, pulls people in opposite directions. On the one hand it calls for ‘progressive’ open-mindedness and respect for cultural others, while on the other hand it demands the acceptance of ‘conservative’ or ‘bad’ behavior, which is deemed non-cosmopolitan and hence ethically troubling. In the Discussion section, we will illuminate the potential implications of this identity struggle for people’s aspirational identity projects and for their encounters with cultural otherness.

#### The Aspirational ‘Humble Visitor’ Identity: Not Confronting the Discriminators

When reflecting on their ideals of ‘cosmopolitan doings,’ interviewees described instances when their efforts at local integration were undermined by people in their host country. Sometimes, their aspiration to engage with locals was met, for example, with indifference, social exclusion, or discrimination, creating a sharp clash between their own ideals around cosmopolitan doings and their real-life experiences of others’ un-cosmopolitan responses. In an earlier quote, Nabil testified to his strong conviction that “integration was up to the foreigner.” However, as a ‘humble visitor,’ he regularly encountered local perceptions full of prejudice:

Their [locals’] knowledge of the world is really limited. They think of [my home country] as a source of diarrhea. It’s omnipresent. Whenever there are problems at work, [the foreigners] are the first to be perceived as being at fault. It’s very unprofessional. […] For example, in the office we always put our coffee mugs in the dishwasher. And sometimes we just put our spoon in the sink. If ever a spoon is in the sink, our secretary always comes to my room first: “Hey Nabil, did you see the spoon?” My God. And then you say… [laughs]. You actually can’t say anything about it, but you feel… And then you really have to be fair. Can you blame her? She’s a very nice 60-year-old Swiss lady, doing secretarial work. The mindset is more ingrained. They don’t even think about whether it’s right or wrong to say things like that. (Interview 29)

In this situation, Nabil experienced what we describe as an identity struggle: on the one hand, he felt unfairly treated and othered by the secretary’s micro-discriminatory accusations; on the other hand, he held on to his cosmopolitan ideal of local integration and ‘fitting in,’ by spinning the story to downplay the secretary’s intentions. Despite feeling hurt, Nabil presented her as a sweet and caring person who was not to blame. In more extreme cases, interviewees reported instances of overt social exclusion at the workplace—cases in which, again, they did not confront the ‘perpetrators’:

There was a major schism in the school. There was a group of older [local] folks who really didn’t like the wave of new people coming in [from abroad], because they felt our research [published in international rather than national journals] was being valued more, which it was, objectively. […] So, every time we had faculty meetings, there were very often implicit attacks on us foreigners. […] And one of my [local] colleagues even said it explicitly: “After the first one of us [foreigners] resigns, they’re gonna have a celebration.” I was the first to resign. I don’t know if they had a celebration, but I knew a group of them would be very happy: “We’ve sent him packing!” That’s why it was super important that I had first received tenure and then left on my own terms, not on anybody else’s. (Interview 25)

Adnan, who shared the reflection quoted above, had together with his family arrived as a young boy in North America as a refugee. He reported that during his childhood he often felt looked down on as a foreigner. A childhood experience of feeling excluded, looked down on, or othered due to a migration background was shared by a handful of other respondents who also had a migration background and/or lived through an international relocation during childhood. Similar to others’ reports, Adnan’s experience of exclusion had fueled his ambition to prove himself to his environment. Always a hard worker, he had been promoted to the position of full professor in his early thirties. His impressive career progression necessitated international relocations to prestigious universities around the globe. Despite his many accomplishments, his success became a new source of exclusion from local colleagues who felt threatened by the influx of international scholars. Reflecting what we characterize as an identity struggle, Adnan seemed torn: although he had spent his life striving for acknowledgment and acceptance from his local communities, as he reported during the interview, he also did not insist on this acknowledgment, as it would have meant confronting colleagues who showed exclusionary behavior toward him. Instead, he chose to resign from his job, where he had failed to integrate into the group of local colleagues—despite, or perhaps because of, his excellent performance.

Interestingly, although interviewees in our study often came across as informed, reflexive, and confident, their cosmopolitan aspirations to fit into a local context also made them vulnerable. Mira, for example, proudly proclaimed that her teaching in global human resource management raised awareness about issues related to diversity, equity, and inclusion, and that she therefore actively participated in intellectual debates on how to advance the agenda of cosmopolitanism. She also recounted a situation, however, in which she suddenly became aware of her own vulnerable position as a foreigner:

I was on the bus going home and there were three Saudis—I could tell by their Arabic dialect that they were from Bahrain. They were sitting in the back talking. I may not speak Arabic well, but I knew that it was all banter. And there was a guy on the bus who turned around and was like, “Speak English in our country! You’re making me very nervous.” And so they became quiet. They happened to get off two stops later, but this man had been quite loud and it had obviously made them feel quite uncomfortable. And part of me wanted to turn around and say, “They were just chatting—like, shut up,” you know. But then another part of me said, “I’m a foreigner, too. I’m not white. I might get targeted, too.” And so I chose to keep quiet. I still think about it today. (Interview 24)

In this episode, Mira experienced a tension between her cosmopolitan aspiration to stand up for others—to protect those publicly attacked or shamed—and her own sense of vulnerability as a foreign-born person of color. Since her English is indistinguishable from that of a native speaker and she also understood the conversation in Arabic, she had felt positioned to speak up; however, she also became acutely aware of her own vulnerabilities, which, despite her successful integration efforts, can be perceived as a marker of difference and inferiority. Like in the two previous cases, Mira thus assumed the role of ‘humble visitor’ choosing not to confront the person exhibiting ‘un-cosmopolitan’ behavior. She openly acknowledged that this moment had induced an unresolved identity struggle by noting that she still “thinks about it today,” despite several years having passed since the incident.

#### The Aspirational ‘Self-Transformed’ Identity: Juggling Self-Other Perceptions

When striving for ‘cosmopolitan becoming’ defined as a transformation toward a better version of one’s self-some interviewees reported that their positive self-transformation had not always been recognized or approved of by the outside world, for example, as described by Mia:

My move to [that country] probably changed me the most. I came back and generally felt that I had changed for the better, into a person I liked more. But I could tell some of my friends were a bit unsure about what to make of this new person. (Interview 13)

This quote illustrates another identity struggle: Mia wanted to embrace a new version of herself, but felt hindered by the opinion of others—she felt that her old friends had not embraced her self-transformation. Since she found this reaction worth mentioning in the interview, we can infer that it interfered with her ability to fully endorse her process of cosmopolitan becoming.

Jukka also described having experienced a cosmopolitan self-transformation throughout his global career, especially during a 4-year assignment at a European Union (EU) institution related to higher education. Although he had personally felt a great sense of professional growth as he broadened his horizons by working abroad with colleagues from all over Europe, he was disappointed by how little this was acknowledged by the colleagues at his old previous job to which he had returned after the assignment:

When I returned […] I expected my boss to see some value in the experience I had gained abroad. I didn’t expect a red carpet, but I did think they would somehow acknowledge my experience and try to use it. But absolutely nothing, total neglect. This is a generalization, but there is a tendency to ignore people with foreign experience—because they’re a danger to you, because they know something you don’t, that you can’t relate to. They can’t handle it, even if they work in a [governmental] organization focused on internationalization [of education]. Maybe it was also because I wasn’t able to demonstrate what I could offer in more concrete terms. I had just hoped they would automatically understand that, since I had worked at the EU level, I knew about these policies and strategies. But the sad thing was: they didn’t even make an effort. (Interview 32)

Although Jukka’s international experience had allowed him to grow professionally, his colleagues had failed to recognize it, leaving him empty-handed despite his personal growth. Nevertheless, his description also reflected a commitment to the cosmopolitan ideal of self-improvement as he refused to fully blame his colleagues for their negligence or jealousy, acknowledging that he also could have done better. This desire for recognition on the one hand and not wanting to ‘show off’ on the other hand arguably created an identity struggle for Jukka.

Similarly, Rahul—a professor from the Global South who had become well-respected in the western world and described himself as a left-wing radical committed to embracing any opportunity to speak up against racism or right-wing ideologies—noted with regret that his process of ‘cosmopolitan becoming’ had not been acknowledged by the outside world. As he put it, he felt his possibilities were limited by his ethnic background: “Yes, my first world passport allows me to travel, but I get busted at immigration. The color of my skin trumps the color of my passport in most places” (Interview 27).

Even though Rahul described enjoying many privileges related to having a “first world passport,” he argued that this did not automatically grant him the status of ‘world citizen’ or recognize his journey of self-transformation. Instead, his aspired cosmopolitan identity is caught up in a struggle between self-image and perception from the outside. As all three examples have indicated, even if global professionals go through a remarkable process of freeing themselves from national boundaries and limited worldviews, the extent to which their self-transformation is recognized by the outside world is still limited.

In the following section, we will discuss the implications of the three identity struggles described above in more detail—namely, of *showing tolerance toward intolerance*, *enduring discrimination in order to fit in,* and *denying one’s aspired self-image to match the perception of others*—all of which are associated with holding on firmly to aspirational cosmopolitan identities that are informed by moral norms. Table 3 provides an overview of these identity struggles and related their cosmopolitan ideals/aspirational identities.

## Discussion

Based on the empirical analysis of 32 life story interviews, this paper has examined how globally mobile professionals narratively construct an ethically informed, aspirational cosmopolitan identity. Furthermore, it has explored how, in light of this moral self-image, such professionals recount and reflect on their everyday interactions with cultural others within their global and local communities. The insights our analysis has garnered contribute to the literatures on everyday cosmopolitanism (e.g., Beck & Sznaider, 2013; Delanty, 2006) cosmopolitan identities (e.g., Grinstein & Wathieu, 2012; Nowicka & Kaweh, 2016; Petroccia & Pitasi, 2022; Skovgaard-Smith & Poulfelt, 2018), and aspirational identities (Koning & Waistell, 2012; Thornborrow & Brown, 2009) in a number of ways. In this section, we will first discuss the implications of our analysis and the conceptual grounding for theorizing cosmopolitanism as an aspirational identity before discussing its ethical implications.

### Conceptualizing Cosmopolitanism as Ethical Aspirations: Being, Doing, and Becoming

Earlier research has approached and conceptualized the notion of cosmopolitanism from different angles, most prominently by viewing it from a political perspective as a set of ethical principles (Nussbaum, 1994, 2010), from a social perspective as an everyday ethical practice (Beck & Sznaider, 2013), or from a critical perspective as an ethical appeal for self-transformation (Delanty, 2006). In this paper, we have brought various elements of these perspectives together by examining cosmopolitanism from the cosmopolitan’s *own* point of view. This is important, for, as Nowicka and Kaweh (2016) note, many studies have tried to assign cosmopolitan qualities to people who are globally mobile without paying much attention to the actual experiences and self-identifications of these groups. Directing attention to their viewpoint helps shed new light on cosmopolitanism as an identity project.

To start, our analysis has shown that globally mobile professionals self-identify as cosmopolitans and that cosmopolitan norms and ideals permeate and pervade their identity discourse as ethical aspirations. Following studies that present the concept as an individual or collective identity project (Ong, 2009; Skovgaard-Smith & Poulfelt, 2018), this paper contributes to cosmopolitan identity studies (e.g., Grinstein & Wathieu, 2012; Nowicka & Kaweh, 2016; Petroccia & Pitasi, 2022; Skovgaard-Smith & Poulfelt, 2018) by describing in detail how the different normative ideals of cosmopolitanism figure as central desires and inspiring points of reference in the cosmopolitan’s aspirational identity talk. Cosmopolitanism thus allows globally mobile professionals to construct a desired or desirable image of themselves through the crafting of an ‘aspirational identity’ (Koning & Waistell, 2012; Thornborrow & Brown, 2009).

Second, and more specifically, viewing cosmopolitanism through a lens of ‘aspirational identities’ has allowed us to both illuminate and integrate key elements of the political, social, and critical perspectives, revealing how distinct normative ideals from each are aspired to by globally mobile professionals. These professionals expressed having: a global mindset (associated with the political ideal of thinking globally; ‘being cosmopolitan’), a commitment to humbly engaging with locals (associated with the social ideal of integrating locally; ‘cosmopolitan doings’), and a desire to remain open to others while pursuing continual self-improvement (associated with the critical ideal of self-transformation; ‘cosmopolitan becoming’). Rather than conceiving of cosmopolitanism as an isolated framework—i.e., as a set of abstract principles, a locally enacted ethic, or a personal project of self-improvement—our analysis has shown that globally mobile professionals draw on all three of these dimensions when constructing their aspirational identities. Together, these resources serve to ‘marshal their dreams and desires’ (Thornborrow & Brown, 2009) as they narratively construct a cosmopolitan sense of self. For studies of aspirational identities outside the field of cosmopolitanism, this threefold distinction between thinking, acting, and transforming may provide useful conceptual grounding and precision for analyzing other forms of aspiration. For example, in Thornborrow and Brown’s (2009) study of how recruits narrate their journey to becoming paratroopers, one can also trace their identity talk for aspirations to ‘think, act, and grow’ into that role—although this insight was not made explicit.

Our analysis further revealed that many statements made by research participants could not be clearly categorized as either expressions of cosmopolitan self-perception (‘being’) or accounts of enacted practice (‘doing’). In fact, most of their statements also reflected a future-oriented dimension, hinting at how or who global professionals aspired to be (‘becoming’). This overlap and entanglement between the different categories, which we have tried to systematize for the sake of analytical clarity and rigor (see Table 3), confirms that these dimensions are best understood as interrelated. Rather than attending to only one of these aspects, the study of cosmopolitan identities requires a lens that acknowledges the closely related and naturally reinforcing nature of actors’ aspirational ‘being,’ ‘doing,’ and ‘becoming.’

This systematic, threefold framework for analyzing cosmopolitanism as an aspirational identity thus contributes a more holistic and comprehensive approach to understanding and investigating cosmopolitan expressions in everyday life, both on a theoretical and methodological level. The scheme provided in Table 3—which shows how the interviewed global professionals’ aspirational cosmopolitan identity projects integrated the three most distinct moral norms described in the literature—may be particularly valuable to other scholars with similar interests as it facilitates engagement with existing theoretical insights without requiring a full review of the literature. This framework may also prove helpful for organizational identity studies more generally, as the distinctions between cosmopolitan ‘being.’ ‘doing’ and ‘becoming’ may have heuristic value beyond the specific context of our paper. For example, we expect that the idea that people aspire to think, act, and grow in a particular ethical spirit can ground future studies that aim for analytical precision in their treatment of individual, organizational, or societal aspirations.

### Cosmopolitan Ideals Meet Everyday Encounters: Identity Struggles

As we listened to the accounts of globally mobile professionals explaining how their cosmopolitan aspirations played out in their everyday lives, we thus observed a recurrent disconnect between their lofty ideals and the realities of practice. Even strict adherence to the cosmopolitan imperatives of showing openness, tolerance, and respect for others (Plage et al., 2017; Skrbiš & Woodward, 2013) did not prevent the experience of friction in participants’ local cross-cultural encounters. Previous studies that have noted such frictions have mostly focused on how cosmopolitans either distance themselves, at least in part, from those in their local communities whom they deem less or anti-cosmopolitan (e.g., Skovgaard-Smith & Poulfelt, 2018) or on how they occasionally display un-cosmopolitan behavior that clashes with their own idealism (e.g., Høy-Petersen & Woodward, 2018). While we also observed similar patterns in the broader scope of our study, this paper has primarily focused on cosmopolitans’ experience of adverse effects when trying to lead a thoroughly ‘cosmopolitan life.’

Interviewees reported that, despite their tireless efforts to become part of a local community, their status as ‘foreigners’ in a host country often made them feel like victims of social exclusion and micro-aggressions. While some reported feeling empowered to speak up in a judgmental environment, others felt ‘silenced’ by these experiences and unable to stand up for themselves or vulnerable others. In addition, their desire to become a ‘world citizen’ was sometimes ignored or denied by people in their local communities, thus interfering with their own identity aspirations. For the sake of harmony, global professionals often decided to ‘swallow’ these experiences, not to confront the perpetrators, or even to leave their prestigious jobs, despite their outstanding performance—or, perhaps, precisely because their achievements created jealousy among local colleagues. Even seemingly neutral ‘administrative procedures’ at border control put some people in the position of feeling like a second-class citizen, with seemingly little agency to effect change.

While the existing literature on identity struggles mostly focuses on the internal struggles of individuals (e.g., Hoyer, 2022; Sveningsson & Alvesson, 2003), this paper has addressed the identity struggles that arise when aspiring world citizens are confronted with inconsistencies between their ethically informed cosmopolitan idealism and their (often not so ideal) real-life experiences with cultural others; between what cosmopolitan aspirants seek and what they may actually attain; between ‘internal’ strivings and ‘external’ impositions and imperfections. In these cases, the cosmopolitan ethic that inspires the globally mobile professional’s sense of self appears caught between the desire for moral self-affirmation and a form of self-denial resulting from having to tolerate what, in their eyes, is the immoral behavior of others.

More generally, our analysis has demonstrated that zooming in on the everyday lives of individuals inspired by ethical ideals provides valuable insight into their lived experiences and identity struggles. While identity research in the field of management and organization studies does tend to rely on self-narratives, it has thus far rarely addressed how these narratives are systematically shaped through social interaction or how identities emerge through ongoing interplay between internal aspirations and external ascriptions (Ybema, 2020). To address this gap, we have shown both how individuals’ self-narratives inform their actions and interactions and how everyday encounters can generate identity tensions.

Specifically, our analysis has shown how and why strict adherence to cosmopolitan ideals can induce identity struggles around the aspirations to think globally (being), act locally (doing), and grow a cosmopolitan mindset (becoming). A first struggle emerged when cosmopolitans’ commitments to global citizenship and tolerance toward others clashed with the intolerance they encountered in their community; a second when their efforts to integrate locally were met with reluctance or exclusion and they decided to endure discrimination in order to fit in; and, finally, a third when they abandoned their aspirations for personal growth in order to better fit the identity positions attributed to them by people in their immediate environment. In each of these situations, aspirants experienced a tension between their attempts to live up to their cosmopolitan ideals and the reality of others’ un-cosmopolitan sentiments or behaviors, which we have summarized as struggles to (a) show tolerance toward intolerance, (b) endure discrimination in order to fit in, and (c) deny one’s aspired self-image to match the perception of others. For an overview, see Table 3.

We further characterize the first identity struggle of showing ‘tolerance toward intolerance’ as ‘paradoxical’ in the sense that it involves a self-contradiction—and the other identity struggles as similarly counterproductive: the globally mobile professional’s ‘morally good intentions’ or even just their eagerness to *be, act, and become* more morally elevated, sometimes provoked adverse effects. In other words, when the cosmopolitan imperative of ‘openness toward the cultural other,’ is fully pursued, it can ironically produce the opposite effect. This became evident when, for example, the interviewed professionals—informed by their cross-cultural experiences—tried to broaden the horizons of their families and friends only to be met with withdrawal or avoidance, which, in turn, further constrained the professionals’ own openness. This paradoxical relationship resembles insights from Morillas and Romani’s (2023) study on critical reflexivity and ideology in which the authors identified a new ideology (which they referred to as *doxa*), epitomized by the maxim ‘diversity is good.’ Because this principle became almost universally associated with positive organizational and societal change, it was rarely, if ever, questioned. As a result, this well-intentioned maxim ultimately reduced critical reflexivity, thereby limiting the agency of those who subscribed to it.

The paradox at the heart of our findings is thus that strict adherence to cosmopolitan norms can actually prevent cosmopolitan aspirants from challenging the ‘un-cosmopolitan’ attitudes of others. We therefore surmise that holding on to cosmopolitan idealism in every situation—by always striving to be, do, and become truly cosmopolitan—may have unforeseen, self-defeating effects: by promoting open-mindedness, cosmopolitanism bites itself in the tail when it diminishes respect for cultural others whom the cosmopolitan deems ‘narrow-minded.’ Cosmopolitan aspirants can also become so committed to the principle of cultural openness that they tolerate being treated in a non-cosmopolitan way themselves, even when this includes obnoxiousness or discrimination, trapped rather than liberated by their own ideals. Hence, the cosmopolitan mission risks falling short: without clear guidance on how to respond to un-cosmopolitan behaviors—or, perhaps more accurately, with conflicting or counterproductive guidance—aspirational identities grounded in cosmopolitan ‘being,’ ‘doing,’ and ‘becoming’ can ‘get stuck’ in practice. This, in turn, can leave individuals in a persistent state of tension between their cosmopolitan ideals and the non-cosmopolitan practices they encounter.

In addition to encountering resistance from others, our analysis also shows that a strong cosmopolitan normativity can lead its aspirants to withdraw themselves. Indeed, in the face of conflicting values, social exclusion, or perceived misunderstandings about their own identity, the globally mobile professionals we interviewed often seemed to double down on their cosmopolitan ideals, clinging even more tightly to their aspirational identity as a ‘global citizen,’ ‘humble visitor,’ or ‘self-transformed cosmopolitan.’ In these moments, their commitment to openness seemed to discourage open dialogue: instead of confronting cross-cultural tensions directly, the cosmopolitan aspirant appears to maintain a superficial politeness while privately grappling with frustration, disappointment, or even emotional injury. This suggests that the pursuit of cosmopolitan ideals may inhibit rather than foster genuine connection—prompting individuals to close themselves off from others while maintaining a sanitized image for the outside world.

Importantly, when cosmopolitan aspirants choose not to confront others who, consciously or not, have provoked their exclusion or self-denial, those others are also denied the opportunity to react, explain, or revise their initial sayings and doings. As a result, cross-cultural interactions may appear amicable on the surface, but not be genuine in the way they are experienced. This raises ethical concerns: rather than encouraging more-ethical outcomes, cosmopolitan ideals may in fact enable, or excuse, (the toleration of) unethical conduct. We therefore argue that the normative imperative to ‘remain open’ in light of un-cosmopolitan behavior can become an obstacle to engaging with conflict in a meaningful way. True openness toward others may instead, we believe, require actually setting boundaries and initiating difficult conversations—opening up to others and communicating on their terms in the face of, for example, intolerance, social exclusion, or ignorance. This, in turn, may eventually help cosmopolitan aspirants move beyond their identity stalemate, allowing individual actors to work through conflict rather than avoid it.

### Implications for Globally Mobile Professionals: Compromised Agency and Wellbeing

The paradoxical identity struggles outlined in this study can be seen as unintended effects that leave globally mobile professionals vulnerable, thereby potentially compromising their overall agency and wellbeing. When these professionals first ‘construct themselves’ as ethical cosmopolitan subjects who make conscious choices about how to interact with cultural others in their everyday encounters, they may feel empowered by a sense of agency, for example, over who they are (or aspire to be), how they act, and whom they hope to become. Yet it is important to recognize that such ‘choices’ are made within existing frameworks that can just as easily restrict as enable actors’ discursive room to maneuver (Thornborrow & Brown, 2009). In our study, we observed that global professionals felt at least partially constrained in their possibilities for responding to anti-cosmopolitan, exclusionary, or even hostile behavior from their immediate environments. By sticking to their cosmopolitan ideals, their agency to act was shaped by an intuited cosmopolitan need to stay open and tolerant toward the cultural other.

In this light, we argue that cosmopolitan ideals and their associated identity struggles can turn individuals into self-disciplining subjects who essentially censor themselves: refraining from speaking up against discrimination in public, avoiding calling out ‘bad behavior’ at work, or resigning rather than confronting the anti-social behavior of local colleagues. We also contend that these subjects regulate their own conduct by uncritically adopting (supposedly) morally elevated subjectivities made available through broader societal discourses, which they then consider to be of their own making. In this way, global professionals might be enacting their (supposedly) desired cosmopolitan self-image through internalized self-discipline in their everyday interactions with cultural others (see also Thornborrow & Brown, 2009). They may be using introspection to continuously assess how well they are ethically ‘being,’ ‘doing,’ and ‘becoming’ cosmopolitan while striving to align themselves more closely with these ideals and correcting for any perceived deviations. In turn, this continuous self-monitoring may feed their sense of self-worth and desire for recognition by others.

While this may paint a rather gloomy picture of cosmopolitanism as an aspirational identity project, we do believe in the value of striving for ethical conduct and the desire to contribute to a better world. What we argue for here, however, is the importance of a more reflexive integration of cosmopolitan ideals, allowing more room for spontaneous deviation and conflict. At the end of the day, these ideals will not serve global professionals or those in their immediate environment if they become new doxas and are strictly adhered to at all costs.

## Concluding Remarks

Processes of globalization arguably necessitate not only a new kind of relational ethics, but also a new understanding both of identity politics (Vaara et al., 2021) and of their ethical implications when dealing with complex interdependencies and encounters with cultural differences. And indeed, as Skovgaard-Smith and Poulfelt (2018) note, socially and relationally accomplished cosmopolitan identities can be considered part of a discursive global sphere of identity politics. This study has suggested that clinging to aspirational cosmopolitan identities can impact the wellbeing of global professionals and complicate their genuine encounters with cultural others, calling for responses that remain open not just to otherness but also to tension and disagreement—responses that are not overly restricted or self-censored by cosmopolitan norms. In this study, we observed that—in the name of embracing otherness—globally mobile professionals accepted intolerance, endured discrimination, and neglected their own desired self-image instead of using these tensions as sources of dialogue. When preoccupied by their cosmopolitan aspiration to show openness toward others, such professionals may fail to acknowledge that cosmopolitanism can also entail embracing the frustrations and misunderstandings inherent to communicating with others, let alone to cross-cultural encounters (see also Nowicka & Kaweh, 2016). While these experiences can be challenging and stressful, they can also be exciting. As noted by Nowicka and Kaweh (2016) in their study on the cosmopolitan identities of UN professionals, constant mobility can also require self-protection from ‘too much’ cultural difference. Hence, we suggest that instead of avoiding or denying the tensions that can arise from living together in a globalized world, cosmopolitan values can serve as a moral horizon that gives meaning to difficult experiences and helps actors make sense of everyday cross-cultural tensions.

We moreover propose that, in order to better care for their own wellbeing, globally mobile professionals should permit themselves more ambivalence and cultural contradictions (Beck, 2002) in their everyday encounters without feeling trapped by the moral dilemmas implied by their own cosmopolitan ideals. Living through daily struggles, not only with oneself but also with others, may in fact be essential to moral development—a process that, when taken seriously, may require suspending cosmopolitan normativity from time to time. Only when conflicts and tensions are accepted as a vital part of living together in a globalized world can people develop agentic avenues for conflict navigation and resolution, thus sustaining a dialogue with cultural others in a polarized world rather than retreating to their like-minded cosmopolitan bubbles.
